# Phage gene expression and host responses lead to infection-dependent costs of CRISPR immunity

**DOI:** 10.1038/s41396-020-00794-w

**Published:** 2020-10-03

**Authors:** Sean Meaden, Loris Capria, Ellinor Alseth, Sylvain Gandon, Ambarish Biswas, Luca Lenzi, Stineke van Houte, Edze R. Westra

**Affiliations:** 1 Biosciences, University of Exeter, Exeter, TR10 9EZ UK; 2 CEFE, Univ Montpellier, CNRS, EPHE, IRD, Univ Paul Valéry Montpellier 3, Montpellier, France; 3 Department of Microbiology and Immunology, University of Otago, Dunedin, New Zealand; 4 Institute of Infection, Veterinary and Ecological Sciences, University of Liverpool, Liverpool, UK

**Keywords:** Bacteriophages, Microbial ecology

## Abstract

CRISPR-Cas immune systems are widespread in bacteria and archaea, but not ubiquitous. Previous work has demonstrated that CRISPR immunity is associated with an infection-induced fitness cost, which may help explain the patchy distribution observed. However, the mechanistic basis of this cost has remained unclear. Using *Pseudomonas aeruginosa* PA14 and its phage DMS3vir as a model, we perform a 30-day evolution experiment under phage mediated selection. We demonstrate that although CRISPR is initially selected for, bacteria carrying mutations in the phage receptor rapidly invade the population following subsequent reinfections. We then test three potential mechanisms for the observed cost of CRISPR: (1) autoimmunity from the acquisition of self-targeting spacers, (2) immunopathology or energetic costs from increased *cas* gene expression and (3) toxicity caused by phage gene expression prior to CRISPR-mediated cleavage. We find that phages can express genes before the immune system clears the infection and that expression of these genes can have a negative effect on host fitness. While infection does not lead to increased expression of *cas* genes, it does cause differential expression of multiple other host processes that may further contribute to the cost of CRISPR immunity. In contrast, we found little support for infection-induced autoimmunological and immunopathological effects. Phage gene expression prior to cleavage of the genome by the CRISPR-Cas immune system is therefore the most parsimonious explanation for the observed phage-induced fitness cost.

## Introduction

CRISPR-Cas immune systems exhibit a widespread, but patchy, distribution in bacteria and archaea [1, 2]. This distribution is indicative of extensive gain and the loss of such adaptive immune systems with high predicted rates of horizontal gene transfer [3–5]. Such gain or loss is likely the result of the balance between potential benefit, i.e., the targeting of phages and deleterious mobile genetic elements (MGEs) against the cost of carriage and expression [6, 7]. Put differently, fitness trade-offs associated with CRISPR immunity may select for alternative resistance strategies and the subsequent loss of CRISPR [8]. Previous work has demonstrated that the cost of CRISPR immunity against lytic phage can be inducible, where the cost is only realised upon phage infection [9, 10]. As a consequence, CRISPR immunity is only favoured over loss or mutation of the phage receptor at a low frequency of infection. In contrast, when infection risk is high, a constitutive defence, mutation of the phage receptor, is selected for since this is associated with a fixed cost that does not change, regardless of the number of encounters with phages [9].

Despite this understanding of the ecological conditions under which different strategies of resistance will be selected for, the mechanistic basis of the inducible cost of CRISPR immunity remains unclear [9, 11]. Using *Pseudomonas aeruginosa* strain PA14 and its lytic phage DMS3vir, we perform a 30-day evolution experiment that supports the previously reported inducible cost of CRISPR immunity and then test hypotheses that have been proposed to explain this cost. Firstly, a cost of autoimmunity, where bacteria acquire self-targeting spacers during infections has been demonstrated in a number of systems and provides a plausible mechanistic basis for a cost of CRISPR immunity [12–15], although diverse mechanisms exist to avoid self-targeting spacer acquisition [16]. Ultimately the relative importance of such self-targeting events at the population level is unknown [17].

Secondly, a cost of enhanced expression of the CRISPR-Cas immune system following infection could also explain the induced fitness cost. Amongst a range of different cues that regulate CRISPR expression (reviewed in [18]), infection can trigger upregulation of *cas* genes and CRISPR arrays [19–21]. Enhanced expression presumably elevates the levels of protection but would likely be associated with a metabolic cost to the host.

The final proposed mechanism behind an inducible cost of CRISPR immunity is that expression of phage-encoded genes may take place prior to clearance of the infection, whereby the phage genome no longer has a deleterious effect on the host, which could be harmful to the host [22]. For example, phage can encode anti-CRISPR proteins which must be expressed before clearance by the immune system in order to function. Anti-CRISPRS are widespread, show a diversity of functions and are expressed rapidly upon infection [23–25]. It is therefore likely that other phage genes may also be expressed prior to clearance. Here we employ a combination of selection experiments, deep sequencing of CRISPR amplicons following phage infection and RNA sequencing approaches to explore the importance of each of these three non-mutually exclusive mechanisms in determining the induced fitness cost of CRISPR-Cas immune systems. Here we use extended evolution experiments that span 30 days under phage selection to examine the frequency of different resistance mechanisms that evolve.

## Materials and methods

### Bacterial strains and phages

The previously described *P. aeruginosa* strains UCBPP-PA14, isogenic mutant Δ*cas1* and phage DMS3vir have been previously described [26] and were used throughout this study. DMS3vir is a modified version of DMS3 that carries a truncated C-repressor, preventing lysogeny [26]. A variant of DMS3vir, known as DMS3mvir, which carries a modified protospacer such that it is targeted by WT PA14, was used for the Δ*cas1* competition experiments and has been described previously [26]. Additionally, a variant of PA14 was used that carries two spacers targeting DMS3vir (BIM2 herein) has been described previously [9] and was used for competition experiments and gene expression profiling. A mutant of DMS3vir with the full *acr* operon, including the *acrIE3* and *aca1* genes and their promoter sequence, removed has previously been described in [27].

### Evolution experiment

The evolution experiment was performed with six replicates by inoculating 6 mL M9 supplemented with 0.2% glucose with ~10^6^ bacteria from an overnight cultures of the WT strain and adding 10^7^ PFU of DMS3vir, followed by incubation at 37 °C while shaking at 180 rpm. Cultures were transferred daily 1:100 for 30 days. The ancestral phage (10^7^ PFU) was added daily to maintain selection for resistance and to constantly induce the cost of CRISPR immunity.

### Immunity and resistance profiling

Bacterial immunity against the ancestral phage was determined as described before [9, 11, 22] by streaking individual clones (24 clones per sample) through ancestral phage DMS3vir and phage DMS3vir carrying the anti-CRISPR F1 (*acrIF1*) gene. Bacterial clones sensitive to both phages were scored as “sensitive”, those resistant to DMS3vir but sensitive to DMS3vir + AcrIF1 were scored as “CRISPR immune”, and bacterial clones resistant to both phages were scored as “surface mutants”. CRISPR-Cas-mediated immunity was further confirmed by PCR (see Table S1 for primers). Surface modification was further confirmed on the basis of colony morphology (since phage DMS3vir is pilus-specific, surface mutants have motility defects, resulting in a modified colony morphology) and a lack of new CRISPR spacers. From these analyses fractions of each phenotype (sensitive, CRISPR immune, surface mutant) were calculated for each replicate experiment.

### Δcas1 competition assays

Relative fitness of bacterial populations with CRISPR-mediated immunity was determined by direct competition with a surface mutant for 3 days. Competitions were carried out using blue–white colony screening between a *lacZ* marked surface mutant and either the WT PA14 or a PA14 mutant that lacks the *cas1* gene. We performed eight replicate experiments of each competition and used a gradient of phage titres (0, 10^6^, 10^7^ and 10^8^ PFU/mL, *n* = 64). The phage used (DMS3mvir) is a mutant of DMS3vir that is targeted by both the WT PA14 and the Δ*cas1* strain.

For competition assays, an overnight culture was grown of each strain and a marked surface mutant. Mixtures were made at a 50:50 ratio and plated at T0. These mixtures were added to 6 mL M9 media supplemented with 0.2% glucose and serially transferred with a 1:100 dilution. Populations were plated after 3 days on LB agar supplemented with 30 μg of X-gal to determine the relative frequencies of the competing strains. Selection coefficients were determined as described in [11].

### Amplicon sequencing

In order to track the changes in genetic diversity of the CRISPR loci, we deep sequenced both CRISPR arrays (CRISPR1 and CRISPR2) from each population described above (see “Evolution experiment”) at 2, 4, 6, 10 and 12 days. Full bacterial genomic DNA was isolated using the Qiagen QIAmp DNA mini kit as per the manufacturer’s protocols. A PCR amplification was performed for both CRISPR arrays (CRISPR1 and CRISPR2, see Tables S1 and S2). PCR reactions contained 5 μl DreamTaq master mix (ThermoScientific, UK), 0.5 μl forward primer, 0.5 μl reverse primer, 1.5 μl MiliQ water, 0.5 μl DMSO, 2 μl template DNA. Sample purity was determined by NanoDrop and DNA concentrations were quantified done using a Qubit fluorometer (ThermoFisher, UK). Two separate CRISPR primer (CRISPR1 and CRISPR2 locus) pairs were designed for two first-round PCRs. Two microliter of DNA was used in a first-round of PCR. The primer design incorporates a recognition sequence to allow a secondary nested PCR process (Table S2). Samples were first purified with Ampure SPRI Beads before entering the second PCR performed to incorporate Illumina adaptor sequences. Samples were purified using Ampure SPRI Beads before being quantified using Qubit and assessed using the Fragment Analyzer. Successfully generated amplicon libraries were taken forward and pooled in equimolar amounts, then size selected on a Pippin prep using a range of 180–600 bps. The quantity and quality of each pool was assessed by Bioanalyzer and subsequently by qPCR using the Illumina Library Quantification Kit from Kapa on a Roche Light Cycler LC480II according to manufacturer’s instructions. Template DNA was denatured according to the protocol described in the Illumina cBot User guide and loaded at 12.5 pM concentration. To help balance the complexity of the amplicon library 15% PhiX was spiked in. The sequencing of each pool was carried out on one lane of an Illumina MiSeq, at 2 × 250 bp paired-end sequencing with v2 chemistry.

### Bioinformatics analysis

#### Sequence quality control

Base-calling and de-multiplexing of indexed reads was performed by CASAVA (version 1.8.2) (Illumina) to produce 30 samples. FASTQ files were trimmed to remove Illumina adaptor sequences using Cutadapt version 1.2.1 [28]. The option “-O 3” was set, so the 3′ end of any reads which matched the adaptor sequence over at least 3 bp was trimmed off. The reads were further trimmed to remove low quality bases, using Sickle version 1.200 with a minimum window quality score of 20. After trimming, reads shorter than 10 bp were removed. The raw reads were subjected to a Cutadapt trimming step to remove PCR primer sequences that could potentially introduce an artificial level of complexity in the samples. To improve base quality in both read pairs, sequencing errors were corrected in both forward and reverse reads using the error-correct module within SPAdes sequence assembler, version 3.1.0 [29]. Read pairs were aligned to produce a single sequence for each pair that would entirely span the amplicon using PEAR (version 0.9.10 [30]). Additionally, sequences with uncalled bases (Ns) were removed. To remove sequences originating from potential PCR primer dimers or from any spurious amplification events, a size selection was applied to each merged sequence set, respectively between 30–140 bp for CRISPR1 and 70–500 bp for CRISPR2. Fragmented PhiX phage genome was added to the sequence library in order to increase the sequence complexity. To remove any “bleed through” of PhiX sequences, each sample was compared with the complete PhiX sequence (GenBank gi9626372) using BLASTN [31]. Sequences matching PhiX (*E* value < 10–5) were filtered out of the dataset.

#### Clustering and diversity metrics

For each dataset, any sequences passing the filters (from any sample) were merged into a single file. This final sequence file, plus its own metadata file describing each sample, was used for the analysis by using a custom pipeline based on QIIME 1.9.0 [32]. Clusters were defined using SWARM [33], using the strictest (default) parameters. This tool aggregates a sequence to a cluster if the sequence shows similarity with any of the sequences already present in that cluster. Importantly, the similarity threshold is not fixed but defined within the dataset. A minimum cluster size filter is applied to retain clusters containing at least two sequences and potential chimeric sequences due to PCR events were discarded as well. To calculate the abundance of each cluster, sequences were then aligned on the centroid sequence identified for each cluster, using a minimum similarity threshold of 99% for the entire length of the sequence using the “usearch_global” function in VSEARCH.

The sequencing depth of all samples was explored using the “Chao 1” [34] richness index plotted as a rarefaction curve. Counts in the cluster abundance tables were repeatedly sub-sampled (rarefied; 33 repetitions) at sampling depths of 1000, 12,000, 22,000, … 150,000. The average Chao 1 value obtained by repeating the test 33 times is assigned as alpha-diversity at that specific number of reads for that sample implemented in Qiime. Because all samples reached a clear asymptote, i.e., no samples were under-sampled with regards to spacer diversity, rarefaction was not applied. An abundance table for each locus was used to estimate the richness and evenness of the samples using the following estimators: total observed sequence variants, Shannon, Simpson, Simpson evenness again conducted with Qiime.

The resulting spacers were extracted from the clustered contigs via CRISPRdetect [35] and mapped to the phage and host genome. CRISPRtarget [36] was also used to identify spacers that mapped to the host genome.

#### Gene expression profiling

Ten replicate cultures of PA14 BIM2 and five of WT were grown overnight in shaken glass vials (180 RPM) of 6 mL LB media at 37 C then standardised to an optical density of 0.5 OD600 (~2 × 10^9^ CFU/mL). Five cultures of BIM2 and five cultures of WT were inoculated with 8 × 10^9^ PFU (in 500 µL) DMS3vir (MOI 0.5). Five cultures of BIM2 remained uninfected as controls. All replicates were vortexed then incubated statically. After 35 min, 1 and 2 h, 1.5 mL of cells were pelleted and snap-frozen at – 80 C. For RNA extraction, 1 mL of TRIzol reagent was added immediately after removal from the freezer. The PureLink RNA mini extraction kit (Invitrogen) was used following instructions for use with TRIzol and the on-column PureLink DNase treatment (Invitrogen). All protocols followed the manufacturer’s instructions except for a substitution of 100 μL BCP instead of chloroform for phase separation. Sample quantities, purities and size distribution were quantified by Qubit, nanodrop and TapeStation respectively. In total, 150 bp libraries were prepared with the TruSeq directional kit, following manufacturer’s instructions, and sequenced on an Illumina NovaSeq at Exeter University sequencing centre.

The resulting reads were quality filtered using Sickle ([37] default settings, version 1.33) with ~6 million read pairs per sample remaining. These were subsequently mapped to the PA14 and DMS3vir reference genomes with bwa ([38] default settings, version 0.7.17). The resulting read alignments were assigned to genomic features and counted using HTSeq (version 0.11.2 [39], using “union” mode, non-unique setting set to “none” and the reverse stranded option). Differential expression analysis was conducted in R using the DESeq2 package [40].

Phage gene expression programme was determined by normalising each gene by maximum and minimum expression levels then averaged across the five replicates of WT PA14 infected by DMS3vir. These profiles were clustered using the “hclust” function in the R “stats” package. A dendrogram was made with the “dendextend” [41]. Sequence data are available from the European Nucleotide Archive under accession number PRJEB31514.

#### Competition assays in the presence of Acr(−) phage

In order to determine the effect of *acrIE3* and *aca1* gene expression on the fitness of immune hosts we competed a WT PA14 strain carrying two spacers that target DMS3vir (BIM2) against a LacZ marked surface mutant in the presence of a phage lacking the full Acr operon and a WT control. While both of hosts are immune to DMS3vir, the surface mutant prevents phage genome injection and therefore any subsequent phage gene expression. Competition experiments were carried out as described above for 24 h under with the addition of 10^5^, 10^7^ and 10^9^ PFU DMS3vir.

#### ProI cloning and assay

The protease I encoding gene from DMS3vir was cloned into the pHERD30T vector under control of an arabinose inducible promoter. The protease I encoding gene was amplified from DMS3vir via PCR and introduced into pHerd30T between an EcoRI and an XbaI site, downstream of an arabinose inducible promoter. Primers used for cloning are available in Table S1. Colonies were selected using a gentamycin marker and blue/white colony screening in a background of DH5-alpha cells (New England Biolabs, UK). The resulting construct was transformed into PA14 and the clone used for assays was verified by Sanger sequencing (University of Sheffield, UK). Optical density was recorded during a 24-h growth curve at 37 C in LB supplemented with 1% arabinose. Growth curves of the clone expressing the protease I encoding gene and an empty vector control were recorded. Growth rate during log-phase was extracted from the growth curves in R (version 3.5.2).

## Results

**Fig. 1:**
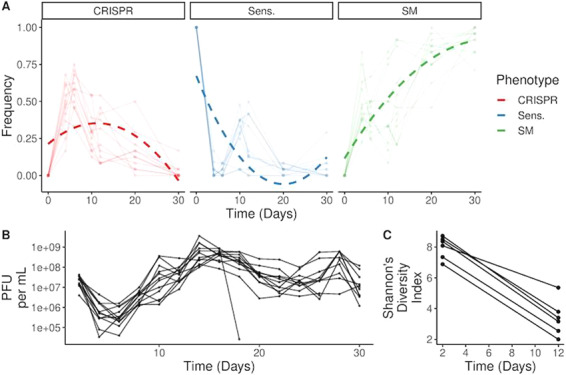
Population dynamics of each bacterial genotype and phage titres. **a** Each point represents the proportion of bacterial phenotypes (*n* = 24 clones per population) that exist in each population. Shaded lines represent individual populations. Dashed lines represent model fit from a GLM that includes a polynomial spline (knots = 2). **b** Lines represent the phage titres present in each population throughout the evolution experiment. **c** Genetic diversity (Shannon’s diversity index) of the CRISPR2 array during the first 12 days of the experiment from amplicon sequence data.

Fitness costs of CRISPR can select for alternative resistance strategies, and the subsequent invasion of bacteria carrying surface-based resistance [9]. However, it has remained unclear what the mechanistic basis of this cost is. We envisaged three possible reasons for this inducible cost: (1) a cost of autoimmunity, where bacteria acquire self-targeting spacers during infections (2) a fitness cost due to enhanced expression of the CRISPR-Cas immune system following infection or (3) due to expression of phage-encoded genes in the period between phage genome injection and CRISPR-mediated clearance of the phage. To explore the first hypothesis, we first carried out an experiment where we infected WT PA14 daily with a high titre of phage DMS3vir, followed by deep sequencing of CRISPR amplicons to measure the frequency of self-targeting spacers. As expected, bacteria initially evolved high levels of CRISPR immunity with corresponding spacer acquisition, which was followed by a consistent increase in the frequency of surface mutants due to the induced fitness costs of CRISPR immunity in the presence of phage (Fig. 1). Deep sequencing of the CRISPR arrays from these bacterial populations revealed a corresponding decline in genetic diversity of CRISPR arrays (Fig. 1). Because of primed spacer acquisition, most spacers target the phage DMS3vir genome in the area proximal to the priming site (position 27847), and the distribution of target sites on the phage genome is similar to that reported in previous studies on this empirical system (Fig. S1 [9, 27]) as well of that of other empirical systems where primed spacer acquisition was observed [42–44]. Arrays carrying self-targeting spacers with either a canonical PAM site (*n* = 3) or a non-canonical PAM site (*n* = 3) to the original host–genome were rare. Moreover, the majority of the spacers with a canonical PAM were only observed in a single sample, consistent with the idea that there is strong selection against a self-targeting spacer. These results therefore suggest that self-targeting is either deleterious or rare. By comparing the number of spacers acquired from the phage with either a canonical or non-canonical PAM site we estimated the background ratio between these acquisition events. By identifying the number of reads that mapped to arrays carrying self-targeting spacers with a non-canonical PAM, we can estimate the proportion of cells that self-target. We found that 0.0068% of cells likely self-target (see Supplementary information for Methods). As PAM sites are a key component of self-target avoidance, this further suggests the phenomenon is rare.

**Fig. 2:**
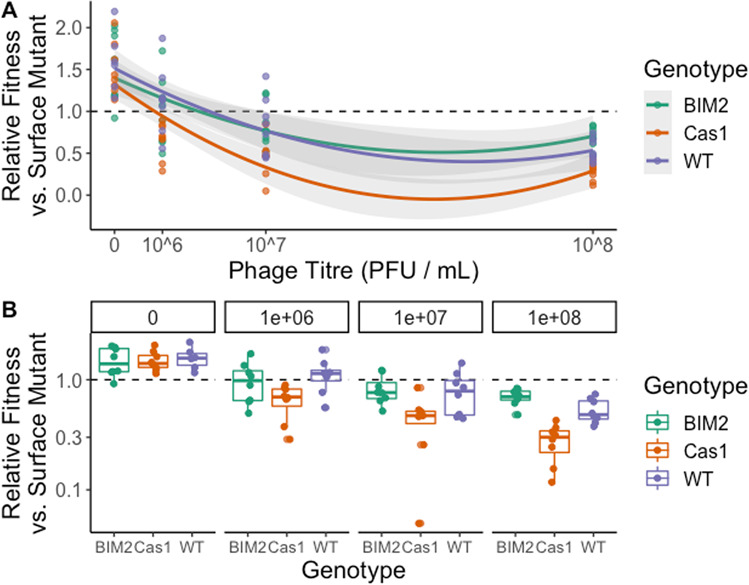
Relative fitness of bacterial populations with CRISPR-mediated immunity at 3 d.p.i. when competing with a surface mutant. Competitions were carried out between a marked surface resistant strain and either the WT PA14 (purple), a mutant PA14 lacking a functional *cas1* (orange) or a WT-derived strain carrying two additional spacers targeting phage DMS3mvir (BIM2; green). Experiments were carried out across a gradient of phage titres, as indicated. The phage used (DMS3mvir) is a mutant of DMS3vir that is targeted by WT PA14. *n* = 8 replicates per genotype per phage titre. **a** Data presented with phage titre as a continuous variable. **b** Data grouped by phage titre to better visualise differences within titres.

To explore whether these self-targeting events have a measurable negative impact on the fitness of the bacteria, we performed a competition experiment between a surface mutant and either WT bacteria with CRISPR immunity, which can acquire self-targeting spacers, or a *cas1* deletion mutant with CRISPR immunity that is unable to acquire novel spacers. This competition was performed across a gradient of titres of DMS3mvir (a mutant of DMS3vir that is targeted by spacer1 in CRISPR array 2 of the PA14Δ*cas1* strain; hence both the surface mutant and competing bacterial genotypes are insensitive to this phage). If the induced fitness cost of CRISPR-Cas immunity was due to the acquisition of self-targeting spacers, we would expect the *cas1* deletion mutant to have a higher relative fitness compared to the WT strain. However, this was not observed; the *cas1* deletion mutant had a lower relative fitness than the WT strain during competition with the surface mutant (GLM, *F*_1,70_ = 17.85, *p* < 0.001, Fig. 2). This is likely due to the evolution of escape phages that carry mutations in their target site, which the *cas1* mutant cannot overcome. Indeed we found a higher number of escape phages evolving in the *cas1* mutant compared to the WT strain at the high titre (10^8^ PFU per mL) (GLM, *F*_1,14_ = 15.5, *p* < 0.01), but this was not observed at a lower phage titre (10^6^ PFU per mL) (GLM, *F*_1,15_ = 2.2, *p* = 0.16). In summary these data show that, even at low phage titres when escape phages are likely to be extremely rare, the benefits of spacer acquisition outweigh any autoimmune effects. Taken together with the deep sequencing data, these results do not imply that the acquisition of self-targeting spacers is the primary driver of the observed phage-induced fitness cost that is associated with CRISPR immunity.

**Fig. 3:**
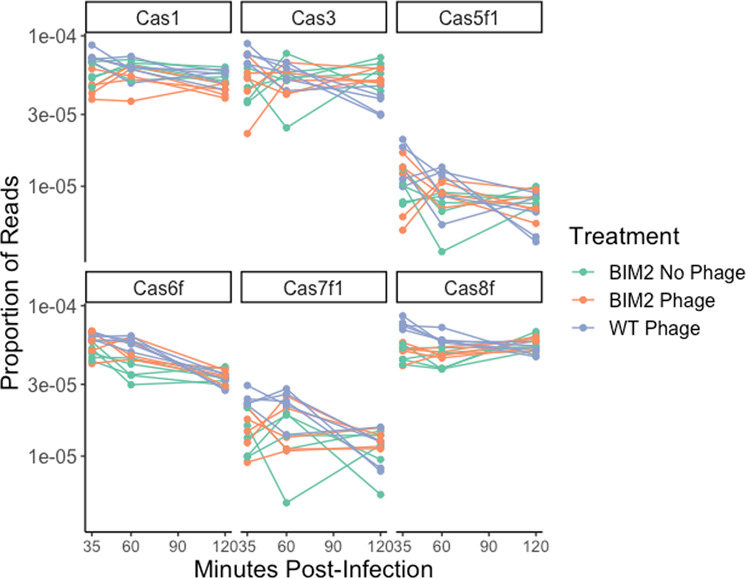
Expression of CRISPR associated genes following infection with 8 × 10^9^ PFU DMS3vir (MOI 0.5) across PA14 WT (purple, *n* = 5), PA14 BIM2 (orange, *n* = 5) and PA14 BIM2 uninfected controls (green, *n* = 5). Samples taken 35, 60 and 120 min post infection.

To explore the second hypothesis, that the induced fitness cost is caused by induction of CRISPR-Cas gene expression, we performed RNAseq of uninfected controls as well as CRISPR-immune bacteria infected with DMS3vir. To avoid evolution of “escape phage”, which carry mutations in the protospacer, we infected a CRISPR-immune clone that carries two spacers targeting DMS3vir (BIM2), and extracted RNA at *t* = 0, 35, 60 and 120 min post infection. BIM2 shows complete immunity to DMS3vir and no phage particles are produced following infection (limit of detection = 200 PFU/mL, data not shown; also see [11]). Differential expression analysis found no evidence that CRISPR-Cas expression is enhanced following infection (Fig. 3). Of all *cas* genes, only *cas1* showed a significant difference, with slightly lower expression in infected BIM2 populations relative to controls (FDR adjusted *p* value = 0.017, log2 fold change = −0.375). These data therefore do not support the idea that the induced cost of CRISPR immunity observed in this experimental system is due to the upregulation of CRISPR-Cas expression.

Interestingly, we identified a number of other host genes that showed significantly different gene expression in the infected BIM2 strain relative to the uninfected controls (Table S3).

Across all three timepoints, genes involved in motility, polyamine breakdown and transport and metabolism were significantly upregulated. The two polyamines associated with phage infection were putrescine and spermidine which are linked to biofilm production (reviewed in [45]). In addition to these polyamines, we found many components of flagellum production to also be upregulated subsequent to phage infection including flagellar rod proteins and the flagellar motor switch. This suggests that bacteria are producing and activating flagella in response to infection. In contrast, we found a significant downregulation of many genes implicated in chemotaxis as well as oxidoreductases and cytochrome c genes and metabolism associated genes. These results suggest a shift in both the metabolism and motility of the bacteria in response to phage infection. Although the communication molecule PQS has previously been shown to be produced during phage infection in order to repel sensitive bacteria from the area [46] we found no significant upregulation of PQS genes. We also observed no obvious effect of time on host gene expression, with the most upregulated genes all occurring at similar levels throughout the experiment.

**Fig. 4:**
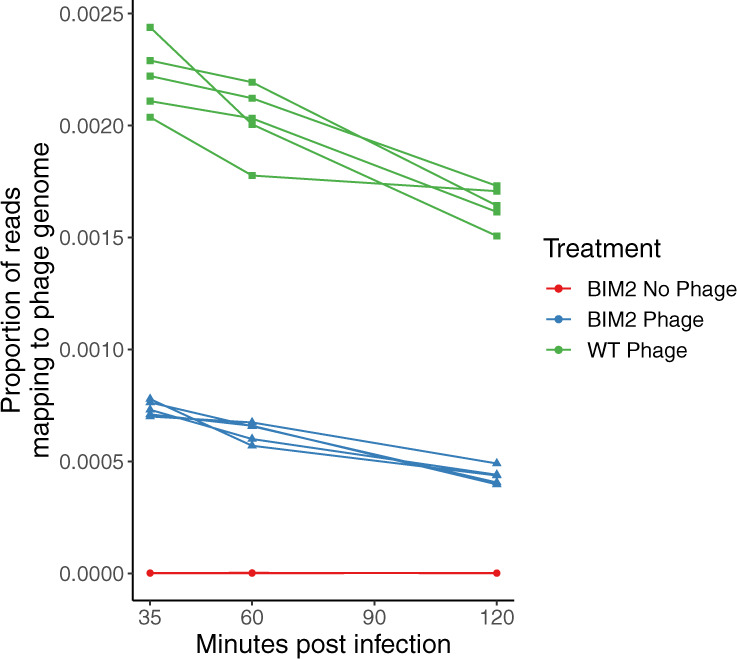
Total phage gene expression following infection with 8 × 10^9^ PFU DMS3vir (MOI 0.5) across PA14 WT (green, *n* = 5), PA14 BIM2 (blue, *n* = 5) and PA14 BIM2 uninfected controls (red, *n* = 5). Samples taken 35, 60 and 120 min post infection.

Finally, to test whether phage gene expression prior to clearance of the infection by the immune system could be responsible for the induced fitness cost, we measured whether phage gene expression could be detected in the infected BIM2 populations. To this end, we mapped all the RNAseq reads to the DMS3vir genome. This revealed significant levels of genome-wide phage gene expression in the BIM2 populations, despite their CRISPR-based immunity. In total, phage expression in the BIM2 populations was around fivefold lower compared to infected WT populations (Figs. 4 and S2). Amongst the expressed phage genes, we identified very high expression levels of the anti-CRISPR (*acrIE3*) and its associated repressor (*aca1*) (Fig. S3), and to a lesser degree, many other genes (Fig S3). *AcrIE3* and *aca1* have previously been reported to be amongst the most strongly expressed genes on the phage DMS3vir genome [25]. This *acrIE3* protein is specific for type I-E CRISPR-Cas systems and does not impact the type I-F CRISPR-Cas system encoded by PA14. Collectively, these data show that the phage is capable of expressing its genes prior to the completion of CRISPR-mediated cleavage.

To test whether expression of the *acrIE3* or *aca1* genes might contribute to the phage-induced fitness cost that is observed in CRISPR-immune bacteria, we next competed the BIM2 strain and the surface mutant in the presence of either WT DMS3vir or a mutant carrying a deletion of the entire *acr* operon. This revealed a similar fitness cost of CRISPR immunity regardless of the phage genotype (GLM, effect of genotype on relative fitness of BIM vs SM, *F*_1,34_ = 2.00, *p* = 0.16), suggesting that the high expression levels of the *acrIE3* and *aca1* genes is not causing the observed induced fitness cost in the host. However, given that many other phage genes are also expressed, albeit at lower levels, the induced cost may well be due to expression of one or more of these other phage genes.

**Fig. 5:**
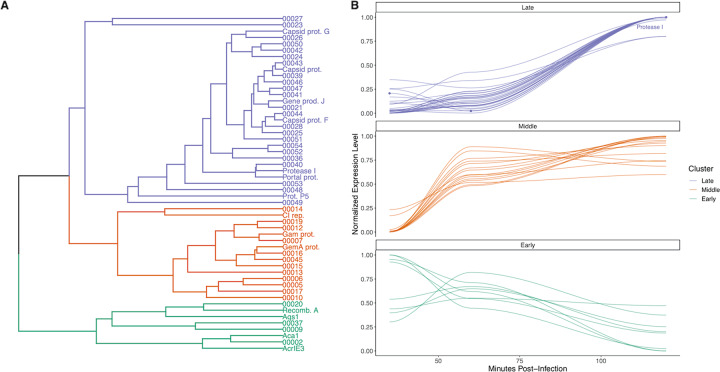
Normalised phage gene expression of DMS3vir infecting WT PA14. Normalised gene expression was averaged across replicates (*n* = 5 per time point) and grouped by hierarchical clustering to identify distinct phases of the transcriptional programme. **a** Dendrogram showing discrete clusters with annotated genes and hypothetical proteins (numbered). **b** Normalised gene expression of each gene through time grouped by each cluster. Lines show local-regression (loess) model fits. Points denote values for Protease I gene expression.

We next clustered the phage genes using their normalised gene expression during infection of WT PA14 (i.e., carries an imperfect CRISPR match which enables priming but not immunity), following the methods of [47] (Fig. 5). We found that consistent with Stanley et al. [25] the *acrIE3* and *aca1* gene are expressed early. We also searched the annotations for potentially damaging genes and identified a protease I gene. Expression of the protease I gene might be particularly costly, since proteolytic activity could conceivably cause cytotoxicity in the cell. This gene is located immediately downstream of the *acrIE3* and *aca1* genes and was found to be expressed both in this experiment as well as a preliminary, independent RNAseq experiment using nanopore sequencing (data not shown). Given that protease I is involved in virion assembly, a deletion mutant would likely not be viable, we therefore cloned and expressed the protease gene in WT *P. aeruginosa* to measure the cost of expression of this gene for the host. This showed that protease I expression reduced cell growth rates by ~13% relative to an empty vector control (Fig. S4, GLM, *F*_1, 46_ = 28.72, *p* < 0.001). This suggests that expression of phage genes prior to phage genome cleavage by the CRISPR-Cas immune system can be costly for the host. Given that this is just one of the many genes expressed by the phage during infection, we argue that phage gene expression prior to clearance of the infection is an important source of the observed phage-induced cost of CRISPR-based immunity.

We developed a mathematical model to explore the competition between different resistant bacteria in the presence or in the absence of phages. Only the CRISPR-immune bacteria are potentially affected by the presence of phages because surface mutants cannot be infected. Our model shows that the dynamics we observe experimentally can be recreated even when self-targeting is absent, provided there is a cost associated with phage infection because of induced immunity or toxicity (see Supplementary information). Importantly, the model predicts that in the absence of a phage-induced cost, bacteria with CRISPR-Cas systems that experience high levels of self-targeting would decline in frequency (Fig. S5C). If the CRISPR-immune bacteria is deficient in spacer acquisition (i.e., *p* = 0) it would not decline in frequency (Fig. S5A). However, this result is inconsistent with our experimental data, which shows that both bacteria with fully functional CRISPR immunity and those deficient in spacer acquisition lose the competition with surface mutants (Fig. 2). Therefore, this model suggests the observed population dynamics are best explained by toxicity effects.

## Discussion

Previous work has demonstrated that CRISPR-immune populations experience infection-dependent costs [9, 10]. Here, we test three hypotheses that might explain the observed infection-induced cost of CRISPR immunity. We find that upregulation of *cas* genes is unlikely to be the cause of this cost. While we cannot rule out increased *cas* protein activity levels, previous work found that plasmid targeting was unaffected by phage pre-infection [48]. Additionally, we find that autoimmunity due to acquisition of self-targeting spacers, although present, is unlikely to be an important source of the observed fitness costs of CRISPR immunity. Although quantifying the true extent of autoimmune effects of self-targeting spacers is difficult as they, by definition, are rapidly lost from the populations, we also observe very low numbers of self-targeting spacers that target sequences with non-canonical PAM. As these spacers would be selected against less strongly it supports the notion that self-targeting spacer acquisitions are rare [12]. Moreover, we see that carrying CRISPR in the absence of phages is not costly [9], that an acquisition-deficient mutant is equally fit to the wild type in the absence of phages (Fig. 2), and there is no detectable difference in CRISPR-Cas expression in the presence or absence of phages (suggesting that rates of self-targeting spacer acquisition would be independent of the presence or absence of phage). This result is also consistent with previous studies that report higher rates of acquisition from MGEs than the host genome, even in the absence of priming [13, 49].

Our data demonstrate that there are high levels of phage gene expression following infection, even with highly effective CRISPR targeting. Phage gene expression likely creates cytotoxic effects for the infected bacterial cells and host gene expression is fundamentally altered. The finding that phages can rapidly express their genes prior to clearance by the CRISPR-Cas immune system is consistent with previous studies [25]. For example, phages that encode *acr* genes can successfully infect bacteria with CRISPR immunity, even though the Acr proteins are produced during the infection cycle. This has selected for extremely rapid and strong expression of the acr gene (*acrIE3*) and its repressor (*aca1*) [25], which we also observed in our RNAseq data. These high expression levels of the *acrIE3* gene, which targets type I-E CRISPR-Cas immune systems and not the type I-F system of the PA14 strain used here, was not associated with a detectable cost. However, we found genome-wide phage gene expression suggesting multiple opportunities for phage mediated damage prior to clearance (Fig. S4). We also found that plasmid-based expression of a phage gene (protease I) showed cytotoxicity and may be cleaving host proteins as well as performing its likely role in capsid packaging [50]. We suggest this may be representative of a widespread cost of phage gene expression and further work is needed to assess the point at which phage DNA no longer has a negative effect on the host cell. Given that, in some cases, the CRISPR-immune system preferentially targets the portion of the phage genome that is injected into the cell first, it appears that preventing phage gene expression as soon as possible is crucial for immunity [51]. Understanding the exact timing of CRISPR interference remains a vital question as the effects of targeting during different parts of the phage replication cycle may be important for the efficiency of immune response. Indeed similar effects may also occur during anti-plasmid immunity, when a plasmid carries genes that are toxic to the host [52]. In addition to the protease we identified a number of host processes that are induced upon phage infection, which may further contribute to the observed infection-induced fitness cost of CRISPR-immune bacteria. Notably the export of spermidine, breakdown of putrescine and construction and activation of flagella may all lead to intrinsic energetic costs. It is unclear why these host processes are induced and their adaptive significance. Given that *Pseudomonas* phages appear unable to replicate on hosts deficient for spermidine production [53], export of spermidine would likely interfere with phage propagation. The upregulation of degradation and export of these polyamines is interesting as polyamine production has been linked to biofilm production and protection from antibiotics–exogenous polyamines may also confer a protective barrier against phages. It is also possible that the change in host processes is a manipulation from the phage, analogous to some eukaryotic viruses that enhance host movement during infection in order to facilitate greater virus dispersal (reviewed in [54]). In this context the phage could induce flagella-based motility in order to disseminate virions to a wider area. An alternative is that flagella production is a host response to leave an area of high relatedness and therefore limit subsequent infections to kin. Such movement away from infected populations has been previously observed in *P. aeruginosa* [46] and motility has been shown to confer a fitness advantage in the presence of phages—although the precise mechanism is unclear [55].

In addition to the effects we observed, there may be additional unknown factors that arise from phage infection. These could include cellular stress from membrane piercing during phage injection, or the costs of producing phage proteins even if they are not cytotoxic. Similarly, Mu-like phages replicate via replicative transposition, which involves integrated into the genome during the lytic cycle, and it is possible that CRISPR targeting of the phage during this step would be costly to the host. Such targeting during lysogenic infection has been observed to result in the upregulation of the SOS response [56] and can lead to the loss of a functional CRISPR system [8]. Although we do not see significant upregulation of the genes associated with this response, we cannot rule out this effect from happening in a sub-population of cells (<1% based on the effect sizes seen in Heussler et al. [56]). As more phage defences are discovered and described it will be fascinating to see how their specific costs and benefits will compare to CRISPR and other well described mechanisms, such as restriction modification. For example, the lag between infection and detection may represent a widespread cost intrinsic to all post-infection defence systems. Our results suggest that although the maintenance of a CRISPR system is likely to be low-cost, the infection-dependent cost is likely to be much higher. Therefore, if the frequency of phage challenge is high, constitutive mechanisms are selected for. Moreover, the interplay between costs and benefits may vary by environment [57]. For example, in our liquid media experiments, spatial structure is absent, or low, which may reduce the requirement for a pilus (the DMS3vir receptor). In a highly structured environment, such trade-offs may differ. More generally, the ecological context will influence how costly it is to lose the pilus, for example coexistence with other bacteria can amplify the costs [58]. In turn, these results may help explain the patchy distribution of CRISPR systems observed in nature as different niches will be associated with varying trade-offs and infection risks. Indeed it is increasingly understood that CRISPR systems may be transferred among bacteria leading to the frequent gain and loss of such systems. This may, in part, be driven by the relative cost–benefit relationship between inducible cost and infection frequency [59].

## Supplementary information

Supplementary Information

Table S3
