# Supplementary material for: Phage gene expression and host responses lead to infection-dependent costs of CRISPR immunity
Source: ISME J. 2020 Oct 3;15(2):534–44. doi: 10.1038/s41396-020-00794-w (PMC8027618; doi:10.1038/s41396-020-00794-w)
Supplement: Supplementary file 1 — Supplementary Information [file 41396_2020_794_MOESM1_ESM.docx]

Supplementary Materials

Figure S1.

Frequency of spacers extracted from unique arrays mapped to the DMS3mvir genome. Lines denote the estimated frequencies across the genome and each strand. High coverage spacers represent spacers that occurred multiple times across different arrays i.e. in conjunction with different spacers. The skew towards position 27847 is a result of a priming spacer that targets this protospacer.


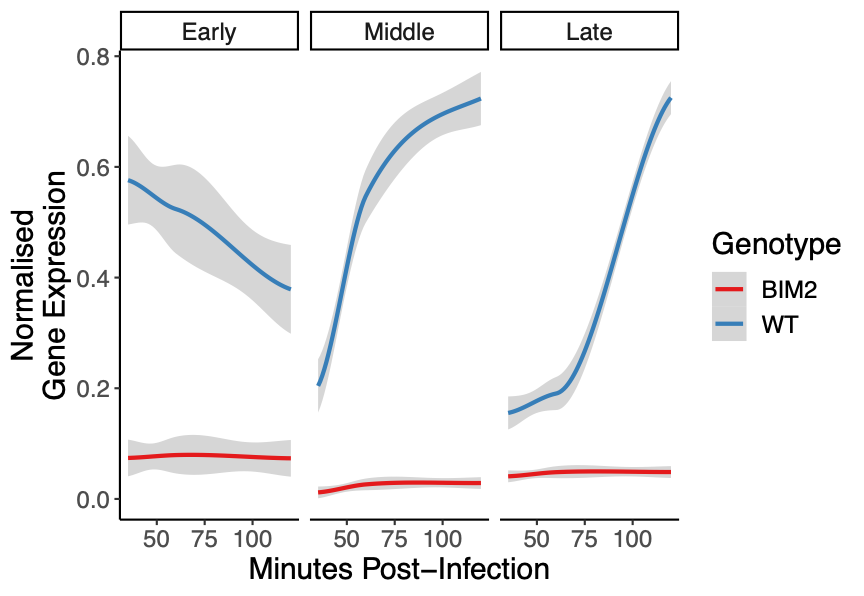


Fig. S2.

Normalised gene expression profiles of each transcriptional phase across host genotypes under phage infection. Lines denote smoothed model fits for all genes in the same category (early/middle/late) across 5 replicates.

Figure S3.

A) Phage gene expression of each annotated phage gene following infection with 8 X 10^9 PFU DMS3vir (MOI 0.5) across PA14 WT (green, n = 5), PA14 BIM2 (blue, n = 5) and PA14 BIM2 uninfected controls (red, n = 5) at 35, 60 and 120 minutes post-infection. B) Gene expression of the *acr* protein (32) and its *aca* repressor (33) across sampling times and treatments.


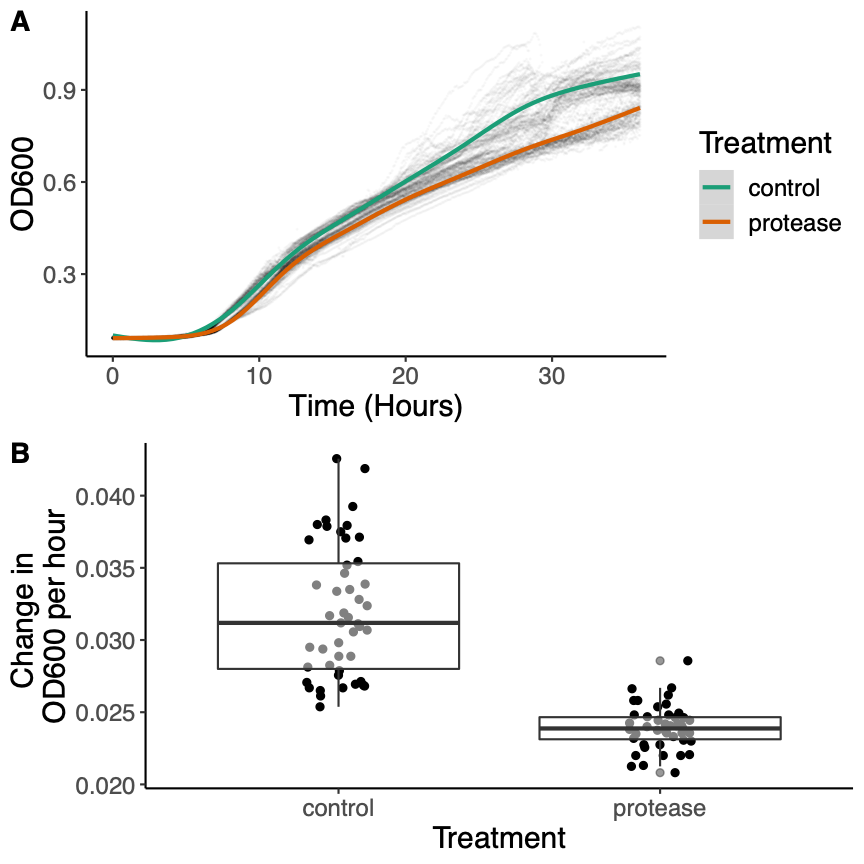


­­­­­­­­

Fig. S4.

Reduction in growth rate when DMS3vir protease (proI) gene is expressed in PA14. A) 36-hour growth curve of empty-vector control and arabinose induced expression of proI. Coloured lines denote mean values, shaded areas represent 95% C.I. and points represent raw data from replicates. B) Change in OD600 during above growth curve between 15 and 20 hours.

Fig. S5.

Dynamics of CRISPR carrying cells (black line) and self-targeting cells (red line) under different fitness cost scenarios. A) No self-targeting cells, no phage-dependent toxicity. B) Self-targeting cells at a frequency of 0.000068, no phage-dependent toxicity. C) 1000-fold increase in self-targeting frequency, no phage-dependent toxicity. D) No self-targeting, phage-dependent toxicity (0.1).

Table S1.

Primers used for cloning ProteaseI, colony array length screening and amplicon sequencing.

| Primer Name | Sequence |
| --- | --- |
| ProI_F | 5’GCCGGAATTCGATGGAAAAGAACCGCCTACTCG |
| ProI_R | 5’GCTCTAGATCAGGCTCCTTTTGCCTTGG |
| colony screen CR1 F | 5’CTAAGCCTTGTACGAAGTCTC |
| colony screen CR1 R | 5’CGCCGAAGGCCAGCGCGCCGGTG |
| colony screen CR2 F | 5’GCCGTCCAGAAGTCACCACCCG |
| colony screen CR2 R | 5’TCAGCAAGTTACGAGACCTCG |
| Amplicon seq CR1 F | 5’GGCGCTGGAGCCCTTGGGGCTTGG |
| Amplicon seq CR1 R | 5’GCGGCTGCCGGTGGTAGCGGGTG |
| Amplicon seq CR2 F | 5’GCTCGACTACTACAACGTCCGGC |
| Amplicon seq CR2 R | 5’GGGTTTCTGGCGGGAAAAACTCGG |

Table S2.

PCR conditions for amplicon sequencing

| PCR round | Temp. | Time | Cycles |
| --- | --- | --- | --- |
| 1 | 95 C | 15 seconds | 10 |
|  | 69 C | 15 seconds |  |
|  | 72 C | 10 min. |  |
| 2 | 95 C | 0 | 15 |
|  | 65 C | 30 seconds |  |
|  | 70 C | 30 seconds |  |
| Extension step | 72 C | 5 seconds | 1 |

Table S3.

Table of significantly up/down regulated genes with annotations (supplementary file).

*Mathematical modeling*

We developed a mathematical model to explore the effects of alternative fitness costs of CRISPR on the dynamics of bacteria in the presence and in the absence of phages. We assess which of the alternative fitness costs we consider in this paper can result in the dynamics we observe. The model includes (1) the cost due to self-targeting (measured by $p$), (2) the cost due to the presence of the phage (measured by $v$) that could result from toxicity due to phage gene expression prior to CRISPR mediated cleavage.

The model tracks the dynamics of three types of bacteria (all bacteria are assumed to be resistant to phages): $B_{0}$, bacteria that do not carry CRISPR, $B_{1}$, bacteria that carry CRISPR without self-targeting, $B_{2}$, bacteria that carry CRISPR with self-targeting. The birth and death rates of the $i$-type bacteria ($i=0, 1 \mathrm{or} 2$) are noted $b_{i}$ and $d_{i}$, respectively. The parameter $p$ measures the proportion that an offspring of $B_{1}$ bacteria self-targets and becomes a $B_{2}$ bacteria. In addition, the parameter $v$ measures the reduction of the birth rate of $B_{1}$ bacteria in the presence of phages. Note that the presence of phages does not affect the growth rate of $B_{0}$ bacteria because bacteria carrying mutations in the phage receptor cannot be infected by the phages. This yields the following set of dynamical equations:

$$\frac{dB_{0}}{dt}=b_{0}B_{0}-d_{0}B_{0}$$

$$\frac{dB_{1}}{dt}=b_{1}\left( 1-v \right)B_{1}-d_{1}B_{1}$$

$$\frac{dB_{2}}{dt}=b_{1}\left( 1-v \right)pB_{1}-d_{2}B_{2}$$

1. *Frequency of self-targeting cells*

The frequency of self-targeting CRISPR cells is noted: $f={B_{2}}/\left( B_{1}+B_{2} \right)$.

The dynamics of $f$ is given by:

$$\frac{df}{dt}=f\left( 1-f \right)\left( \left( d_{1}-d_{2} \right)-b_{1}\left( 1-\frac{p}{f} \right)\left( 1-v \right) \right)$$

The equilibrium frequency of self-targeting cells among CRISPR carrying cells is thus:

$$f_{equilibrium}= \frac{p b_{1} \left( 1-v \right)}{b_{1} \left( 1-v \right) - \left( d_{1}-d_{2} \right)}$$

With this model we find that if the death rate of CRISPR cells (either self-targeting or non-self-targeting) is equal (i.e. $d_{1}=d_{2}$), then the equilibrium frequency of self-targeting cells is equal to the probability of CRISPR cell becoming self-targeting (i.e. $f_{equilibrium}=p$). If the death rate of self-targeting cells is greater than that of non-self-targeting cells (i.e. $d_{2}>d_{1}$) then the equilibrium frequency tends towards zero.

1. *Costs of CRISPR*

The cost of CRISPR is measured by the competition between CRISPR ($B_{1}+B_{2}$) and non-CRISPR ($B_{0}$) cells. Tracking the dynamics of CRISPR carrying cells allows us to measure the costs of CRISPR in our system.

The frequency of CRISPR carrying cells is noted: $g=\left( B_{1}+B_{2} \right)/\left( B_{0}+B_{1}+B_{2} \right)$.

The dynamics of $g$ is given by:

$$\frac{dg}{dt}= \underset{genetic variance}{\underbrace{g\left( 1-g \right)}}\underset{selection coefficient on CRISPR}{\underbrace{\left( \left( d_{0}-d_{1}\left( 1-f \right)-d_{2}f \right)+b_{1}\left( 1-f \right)\left( 1-v \right)-b_{0} \right)}}$$

If we assume that the death rate of CRISPR and non-CRISPR cells is equal ($d_{0}=d_{1}$), but that the death rate of self-targeting cells is greater than that of non-self-targeting cells ($d_{2}>d_{1}$) then frequency $f$ of self-targeting cells falls to 0 (see above).

Therefore, the quantity $b_{1}\left( 1-v \right)-b_{0}$ (i.e. the difference in fecundity between CRISPR and non-CRISPR carrying cells) measures the fitness cost of CRISPR.

We find that CRISPR carrying cells can win when in the absence of phage, and provided $b_{1}>b_{0}$. But when we add phages this adds the cost $v$ because of induced immunity and/or toxicity.

1. *Estimating the required parameters to recreate observed dynamics*

We next plot the frequency $g$ of CRISPR bacteria (black line) and the frequency $f$ of self-targeting CRISPR bacteria (red) under different parameter values. We start with a probability of self-targeting $p=0.000068$, based on the frequency of self-targeting spacers we identified in our sequence data. We use a death rate of self-targeting cells $d_{2}=2$ (which is 4X that of $d_{0}=d_{1}=0.5$) but without the phage-dependent fitness cost ($v=0$).

We next increase the probability of CRISPR cells self-targeting by 1000X:

This more closely resembles the population dynamic data in our experiment. Therefore, in the absence of toxicity, our estimates of self-targeting would need to be 1000X fold higher than the observed rate in order to recreate the observed population dynamics.

In contrast, in the absence of self-targeting (i.e. $p=0)$, small increases in toxicity can reproduce a similar dynamic. For example, a phage dependent cost $v=0.1$ yields:

Finally, when we include both a self-targeting probability $p=0.1$ and a phage-dependent cost $v=0.2$ we find a more rapid decline in CRISPR bacteria that leads to extinction after 14 days, suggesting that the combined effects of both phenomena would rapidly drive CRISPR cells extinct.

*PAM analysis*

Methods

Spacers that target protospacers with a non-canonical PAM site do not result in effective targeting (Deveau et al. 2008; Westra et al. 2013; Rollins et al. 2015). Therefore these self-targeting spacers should not be selected against if their targets are flanked by sequences that deviate from the GG PAM sequence. While the majority of spacers are selected by the integration machinery to target sequences flanked by a PAM, rare mistakes result in a proportion of spacers that target sequences with a mutated, or – perhaps more accurately – “non-canonical”, PAM (Stern et al. 2010). By estimating the ratio of spacers that target the phage with either a canonical PAM or a non-canonical PAM, we can derive the background rate of these acquisition events. By then applying this ratio to the spacers that self-target with a non-canonical PAM we can estimate the number of spacers with a canonical PAM that we should expect to see.

In order to determine the background ratio of canonical/non-canonical PAM acquisitions, we used multi-spacer arrays where the target of the first acquired spacer (defined as furthest from the leader sequence) had a canonical PAM. Arrays where this was not the case were excluded as they would likely be selected against due to lack of immunity against the phage. We then focused on the subsequent spacer acquisitions in the array as these will be less subject to selection effects. We also only use samples from a single timepoint (Day 2) to reduce further selection effects. Because of these criteria, we focus on the CRISPR2 arrays since we did not identify CRISPR1 arrays with multi-spacer arrays at this timepoint.

After identifying arrays and matching the criteria above we extracted the appropriate spacers and mapped them to the phage genome. We then extracted the flanking PAM sequences and determined the background ratio of canonical/non-canonical PAM sites.

Spacers from were extracted using CRISPRdetect (Biswas et al. 2013). Duplicate spacer sequences were removed and the resulting spacers were mapped to both the host and phage genomes. Adjacent PAM regions were extracted using the ‘flank’ command in bedtools (Quinlan et al. 2010).

Results

Overall we identified 6 self-targeting spacers, of which 3 we were in the CRISPR1 array (X2 non-canonical PAM, X1 canonical PAM) and 3 were in the CRISPR2 array (X1 non-canonical PAM, X2 canonical PAM). We found 47815 protospacers that had a canonical PAM, while 1271 had a non-canonical PAM. This gives a ratio of 37.6 for spacer targets with non-canonical PAM over those with canonical PAM, all of which target the phage genome. Given our selection criteria, we found only a single protospacer with a non-canonical PAM that self-targeted. Assuming the same ratio, we should expect to see ~38 spacers with a canonical PAM. To generate a more accurate estimation of the frequency of self-targeting, we should take the abundance of the arrays into account. To do this, we first calculate the proportion of CRISPR reads that contain the self-targeting spacer with the non-canonical PAM. Given there were only 2 reads that contain this self-targeting spacer, and the total read count is 1095805, the proportion of self-targeting from a non-canonical PAM is 2/1095805=1.8x10^-6. Now we multiply this by the ratio of targets with canonical/non-canonical PAM. This gives a frequency of 0.000068 of self-targeting events. We then used this estimate in our mathematical modelling.

References

Deveau, H., Barrangou, R., Garneau, J. E., Labonté, J., Fremaux, C., Boyaval, P., ... & Moineau, S. (2008). Phage response to CRISPR-encoded resistance in Streptococcus thermophilus. *Journal of bacteriology*, *190*(4), 1390-1400.

Westra, E. R., Semenova, E., Datsenko, K. A., Jackson, R. N., Wiedenheft, B., Severinov, K., & Brouns, S. J. (2013). Type IE CRISPR-cas systems discriminate target from non-target DNA through base pairing-independent PAM recognition. *PLoS Genet*, *9*(9), e1003742.

Rollins, M. F., Schuman, J. T., Paulus, K., Bukhari, H. S., & Wiedenheft, B. (2015). Mechanism of foreign DNA recognition by a CRISPR RNA-guided surveillance complex from Pseudomonas aeruginosa. *Nucleic acids research*, *43*(4), 2216-2222.

Stern, A., Keren, L., Wurtzel, O., Amitai, G., & Sorek, R. (2010). Self-targeting by CRISPR: gene regulation or autoimmunity?. *Trends in genetics*, *26*(8), 335-340.

Biswas, A., Staals, R. H., Morales, S. E., Fineran, P. C., & Brown, C. M. (2016). CRISPRDetect: A flexible algorithm to define CRISPR arrays. *BMC genomics*, *17*(1), 1-14.

Quinlan, A. R., & Hall, I. M. (2010). BEDTools: a flexible suite of utilities for comparing genomic features. *Bioinformatics*, *26*(6), 841-842.
