# Supplementary material for: Phage gene expression and host responses lead to infection-dependent costs of CRISPR immunity
Source: ISME J. 2020 Oct 3;15(2):534–44. doi: 10.1038/s41396-020-00794-w (PMC8027618; doi:10.1038/s41396-020-00794-w)
Supplement: Supplementary file 2 — Table S3 [file 41396_2020_794_MOESM2_ESM.pdf]

| ID | log2FoldChange | padj | gene | time | Uniprot | Notes | Class | pa14_locus_tag |
|---|---|---|---|---|---|---|---|---|
| BPAMKIAM_ | -1.020613641 | 8.61E-19 | NA | 60 | NA | NA | NA | PA14_00080 |
| BPAMKIAM_ | -1.004907666 | 5.53E-19 | NA | 120 | NA | NA | NA | PA14_00080 |
| BPAMKIAM_ | -1.350052836 | 0.005076332 | cheB | 120 | Q9I6V9 | chemotaxis signal transduction pathway | motility | PA14_02180 |
| BPAMKIAM_ | -1.175495368 | 0.000423359 | cheD | 120 | NA | chemotaxis signal transduction pathway | motility | PA14_02190 |
| BPAMKIAM_ | -1.295630899 | 2.10E-19 | cheR | 120 | P07801 | chemotaxis protein methyltransferase | motility | PA14_02200 |
| BPAMKIAM_ | -1.195188553 | 3.77E-27 | NA | 120 | NA | NA | NA | PA14_02220 |
| BPAMKIAM_ | -1.382317743 | 6.91E-20 | cheW | 120 | P0A964 | chemotaxis | motility | PA14_02230 |
| BPAMKIAM_ | -1.484476962 | 4.25E-26 | NA | 120 | NA | NA | NA | PA14_02250 |
| BPAMKIAM_ | -1.108727148 | 4.49E-24 | NA | 60 | NA | NA | NA | PA14_02250 |
| BPAMKIAM_ | -1.089001479 | 9.87E-26 | cheY | 120 | P0AE67 | chemotaxis protein; sensory regulation of flagellum | motility | PA14_02260 |
| BPAMKIAM_ | -1.287054304 | 9.87E-26 | NA | 120 | NA | NA | NA | PA14_02270 |
| BPAMKIAM_ | -1.247839255 | 1.56E-06 | NA | 60 | NA | NA | NA | PA14_02270 |
| BPAMKIAM_ | -1.323468868 | 0.000232877 | pntA | 120 | P07001 | NADPH regenration; proton transmembrane transport | transport | PA14_02460 |
| BPAMKIAM_ | -1.132404706 | 0.000986538 | pntA | 60 | P07001 | NADPH regenration; proton transmembrane transport | transport | PA14_02460 |
| BPAMKIAM_ | 1.463302059 | 3.34E-18 | cysA | 120 | P16676 | sulfate transport | transport | PA14_03650 |
| BPAMKIAM_ | 1.274837984 | 5.90E-08 | cysA | 60 | P16676 | sulfate transport | transport | PA14_03650 |
| BPAMKIAM_ | 1.30541315 | 7.21E-06 | cysW | 120 | P0AEB0 | sulfate transport | transport | PA14_03670 |
| BPAMKIAM_ | 1.152831095 | 2.13E-05 | ygiW | 120 | P0ADU5 | biofilm formation; ceullar response | signalling | PA14_04180 |
| BPAMKIAM_ | 1.39824038 | 5.67E-08 | ygiW | 60 | P0ADU5 | biofilm formation; ceullar response | signalling | PA14_04180 |
| BPAMKIAM_ | -1.291015796 | 6.88E-07 | dht | 60 | Q9I676 | dihydropyriminase | metabolism | PA14_05770 |
| BPAMKIAM_ | -1.170191366 | 2.50E-05 | dht | 120 | Q9I676 | dihydropyriminase | metabolism | PA14_05770 |
| BPAMKIAM_ | 2.034594708 | 5.30E-38 | yfdE | 120 | P76518 | acetyl coenzyme A | metabolism | PA14_05820 |
| BPAMKIAM_ | 1.589101759 | 2.01E-29 | mmgC | 60 | P45857 | acyl-coA dehydrogenase; sporulation | metabolism | PA14_05840 |
| BPAMKIAM_ | 3.129428403 | 1.70E-161 | mmgC | 120 | P45857 | acyl-coA dehydrogenase; sporulation | metabolism | PA14_05840 |
| BPAMKIAM_ | 1.261713032 | 0.000986538 | norB | 60 | Q59647 | nitric oxide reducatse | metabolism | PA14_06830 |
| BPAMKIAM_ | 1.326300564 | 0.001665032 | NA | 60 | NA | NA | NA | PA14_06840 |
| BPAMKIAM_ | 1.101798382 | 0.000104258 | NA | 60 | NA | NA | NA | PA14_06860 |
| BPAMKIAM_ | 1.251241295 | 4.84E-08 | NA | 120 | NA | NA | NA | PA14_06860 |
| BPAMKIAM_ | 1.822416308 | 0.000892989 | NA | 120 | NA | NA | NA | PA14_06940 |
| BPAMKIAM_ | 3.583346214 | 1.11E-166 | puuB | 120 | P37906 | putrescine breakdown | metabolism | PA14_06960 |
| BPAMKIAM_ | 3.068314182 | 8.47E-27 | puuB | 35 | P37906 | putrescine breakdown | metabolism | PA14_06960 |
| BPAMKIAM_ | 4.430895213 | 1.27E-82 | puuB | 60 | P37906 | putrescine breakdown | metabolism | PA14_06960 |
| BPAMKIAM_ | 3.484844334 | 2.72E-32 | NA | 60 | NA | NA | NA | PA14_06970 |
| BPAMKIAM_ | 1.66046939 | 2.44E-05 | NA | 35 | NA | NA | NA | PA14_06970 |
| BPAMKIAM_ | 3.624704908 | 3.69E-86 | NA | 120 | NA | NA | NA | PA14_06970 |
| BPAMKIAM_ | -3.127269925 | 3.83E-85 | paiB | 120 | P21341 | protease synthase; sporulation | metabolism | PA14_09790 |
| BPAMKIAM_ | -4.667730889 | 8.48E-09 | NA | 120 | NA | NA | NA | PA14_09810 |
| BPAMKIAM_ | 1.064248358 | 2.81E-05 | NA | 120 | NA | NA | NA | PA14_11790 |
| BPAMKIAM_ | 1.127374744 | 6.66E-20 | ald | 60 | Q9FDS1 | nicotine catabolism | metabolism | NA |
| BPAMKIAM_ | 1.198568914 | 2.10E-19 | ald | 120 | Q9FDS1 | nicotine catabolism | metabolism | NA |
| BPAMKIAM_ | 1.136604033 | 7.46E-14 | ald | 35 | Q9FDS1 | nicotine catabolism | metabolism | NA |
| BPAMKIAM_ | 1.94263108 | 5.46E-05 | NA | 120 | NA | NA | NA | PA14_40820 |
| BPAMKIAM_ | 1.75938133 | 0.000182681 | moaB | 60 | P0AEZ9 | molybdopterin biosynthesis | metabolism | PA14_13260 |
| BPAMKIAM_ | 1.51738951 | 0.011160926 | moaB | 35 | P0AEZ9 | molybdopterin biosynthesis | metabolism | PA14_13260 |
| BPAMKIAM_ | 1.8664432 | 0.006555852 | NA | 120 | NA | NA | NA | NA |
| BPAMKIAM_ | 2.413147737 | 0.000391149 | NA | 35 | NA | NA | NA | NA |
| BPAMKIAM_ | 2.373435002 | 2.04E-06 | NA | 60 | NA | NA | NA | NA |

| | | | | | | | | |
|---|---|---|---|---|---|---|---|---|
| BPAMKIAM_ | 2.296220886 | 9.34E-06 | narK | 60 | P10903 | nitrate transporter | metabolism | PA14_13770 |
| BPAMKIAM_ | 1.976165361 | 2.30E-06 | narK | 120 | P10903 | nitrate transporter | metabolism | PA14_13770 |
| BPAMKIAM_ | 2.16366339 | 1.25E-06 | narK | 35 | P10903 | nitrate transporter | metabolism | PA14_13770 |
| BPAMKIAM_ | 1.556581059 | 7.47E-05 | narG | 120 | P09152 | nitrate metabolism | metabolism | PA14_13780 |
| BPAMKIAM_ | 2.396664768 | 1.93E-08 | narG | 60 | P09152 | nitrate metabolism | metabolism | PA14_13780 |
| BPAMKIAM_ | 2.091021915 | 3.31E-06 | narG | 35 | P09152 | nitrate metabolism | metabolism | PA14_13780 |
| BPAMKIAM_ | 1.471038285 | 0.000602092 | narY | 60 | P19318 | nitrate reducatse | metabolism | PA14_13800 |
| BPAMKIAM_ | 1.505981983 | 9.91E-07 | narY | 120 | P19318 | nitrate reducatse | metabolism | PA14_13800 |
| BPAMKIAM_ | 2.070110242 | 1.61E-08 | narV | 120 | P0AF32 | nitrate reducatse | metabolism | PA14_13830 |
| BPAMKIAM_ | 1.556588698 | 0.006072436 | narV | 60 | P0AF32 | nitrate reducatse | metabolism | PA14_13830 |
| BPAMKIAM_ | 2.184216909 | 0.010191371 | surA | 60 | NA | outermembrane assemly; required for pilus formation | transport | PA14_13840 |
| BPAMKIAM_ | 2.063479155 | 1.26E-05 | surA | 120 | NA | outermembrane assemly; required for pilus formation | transport | PA14_13840 |
| BPAMKIAM_ | 1.689073226 | 0.000334861 | moaA | 120 | Q44118 | molybdopterin biosynthesis | metabolism | PA14_13850 |
| BPAMKIAM_ | -1.43161934 | 3.31E-06 | NA | 35 | NA | NA | NA | PA14_14420 |
| BPAMKIAM_ | -1.652940974 | 1.66E-14 | NA | 60 | NA | NA | NA | PA14_14420 |
| BPAMKIAM_ | -1.057180464 | 1.63E-06 | NA | 120 | NA | NA | NA | PA14_14420 |
| BPAMKIAM_ | -1.027239824 | 5.48E-21 | NA | 35 | NA | NA | NA | PA14_16020 |
| BPAMKIAM_ | -1.03309146 | 0.000780744 | NA | 120 | NA | NA | NA | PA14_16100 |
| BPAMKIAM_ | 1.109640768 | 0.003683592 | yaeQ | 120 | NA | unknown | NA | PA14_16210 |
| BPAMKIAM_ | 1.382636808 | 1.22E-10 | yodB | 120 | P76345 | transcriptional regulator; degradation of aromatic compounds | metabolism | PA14_18060 |
| BPAMKIAM_ | 1.431345134 | 6.81E-08 | NA | 120 | NA | NA | NA | PA14_18680 |
| BPAMKIAM_ | -1.054461308 | 0.038791867 | NA | 120 | NA | NA | NA | PA14_18820 |
| BPAMKIAM_ | 1.581227634 | 8.25E-13 | NA | 60 | NA | NA | NA | PA14_20460 |
| BPAMKIAM_ | 1.064680167 | 1.51E-08 | NA | 120 | NA | NA | NA | PA14_20460 |
| BPAMKIAM_ | 1.46767688 | 1.43E-06 | NA | 35 | NA | NA | NA | PA14_20460 |
| BPAMKIAM_ | 1.618412106 | 1.84E-10 | NA | 60 | NA | NA | NA | PA14_20470 |
| BPAMKIAM_ | 1.503655555 | 6.44E-10 | NA | 35 | NA | NA | NA | PA14_20470 |
| BPAMKIAM_ | 1.459067278 | 1.45E-10 | NA | 35 | NA | NA | NA | PA14_20480 |
| BPAMKIAM_ | 1.040518713 | 7.80E-06 | NA | 60 | NA | NA | NA | PA14_20480 |
| BPAMKIAM_ | 1.622110375 | 1.15E-10 | NA | 35 | NA | NA | NA | PA14_20740 |
| BPAMKIAM_ | 1.335817868 | 3.15E-13 | fecA | 120 | P13036 | siderophore uptake; signalling receptor | signalling | NA |
| BPAMKIAM_ | 1.226020664 | 5.30E-05 | NA | 120 | NA | NA | NA | NA |
| BPAMKIAM_ | -1.167187208 | 0.005575216 | NA | 120 | NA | NA | NA | PA14_21920 |
| BPAMKIAM_ | -1.531956708 | 3.43E-12 | yjcH | 120 | P0AF54 | membrane protein | transport | PA14_22340 |
| BPAMKIAM_ | -1.699905004 | 4.22E-06 | yjcH | 60 | P0AF54 | membrane protein | transport | PA14_22340 |
| BPAMKIAM_ | 1.077137174 | 0.000584158 | NA | 120 | NA | NA | NA | PA14_22720 |
| BPAMKIAM_ | 1.331790303 | 0.000267114 | sasA | 60 | NA | adaptive response; signal transduction | signalling | PA14_22730 |
| BPAMKIAM_ | 1.815233833 | 2.41E-09 | sasA | 120 | NA | adaptive response; signal transduction | signalling | PA14_22730 |
| BPAMKIAM_ | 2.006893878 | 1.07E-19 | NA | 120 | NA | NA | NA | PA14_22740 |
| BPAMKIAM_ | 1.6618377 | 5.03E-11 | NA | 60 | NA | NA | NA | PA14_22740 |
| BPAMKIAM_ | 1.186951658 | 0.00896385 | NA | 35 | NA | NA | NA | PA14_22740 |
| BPAMKIAM_ | 1.459508248 | 0.000786816 | pgl | 120 | P74618 | glucose metabolism | metabolism | PA14_23080 |
| BPAMKIAM_ | 1.251608477 | 0.010054773 | NA | 120 | NA | NA | NA | NA |
| BPAMKIAM_ | 1.197355066 | 3.26E-05 | NA | 35 | NA | NA | NA | PA14_25820 |
| BPAMKIAM_ | 1.326409984 | 0.006388049 | NA | 35 | NA | NA | NA | PA14_25830 |
| BPAMKIAM_ | 1.460821148 | 0.000460888 | NA | 60 | NA | NA | NA | PA14_25830 |
| BPAMKIAM_ | 1.603434499 | 0.014984759 | NA | 35 | NA | NA | NA | NA |
| BPAMKIAM_ | 1.020078382 | 0.033864537 | NA | 120 | NA | NA | NA | NA |

| | | | | | | | | | |
|---|---|---|---|---|---|---|---|---|---|
| BPAMKIAM_ | 1.325898714 | 3.29E-20 | NA | 35 | NA | NA | NA | PA14_28600 |
| BPAMKIAM_ | -1.041190412 | 1.02E-12 | NA | 120 | NA | NA | NA | PA14_29330 |
| BPAMKIAM_ | 1.096828632 | 0.012490521 | pfeS | 120 | Q04804 | sensor protein | signalling | PA14_29360 |
| BPAMKIAM_ | -1.197541843 | 7.97E-15 | lecA | 120 | Q05097 | carbohydrate binding; cell-cell adhesion | metabolism | PA14_31290 |
| BPAMKIAM_ | -1.139862981 | 0.00154533 | lecA | 60 | Q05097 | carbohydrate binding; cell-cell adhesion | metabolism | PA14_31290 |
| BPAMKIAM_ | -1.023371774 | 2.01E-14 | sqr | 60 | O67931 | quinone binding; nucleotide binding | nucleotide_binding | PA14_31350 |
| BPAMKIAM_ | -1.24472775 | 8.17E-24 | sqr | 120 | O67931 | quinone binding; nucleotide binding | nucleotide_binding | PA14_31350 |
| BPAMKIAM_ | -1.044011371 | 3.69E-07 | NA | 60 | NA | NA | NA | PA14_31450 |
| BPAMKIAM_ | -1.070742159 | 4.76E-07 | acsA | 60 | P39062 | acetyl coenzyme A | metabolism | PA14_31500 |
| BPAMKIAM_ | 1.310641316 | 3.43E-11 | mdtA | 120 | P76397 | transmembrane transport; xenobiotic efflux | transport | PA14_31870 |
| BPAMKIAM_ | 1.158471934 | 2.42E-18 | mdtB | 120 | B7M458 | transmembrane transport; xenobiotic efflux | transport | NA |
| BPAMKIAM_ | 1.168281676 | 1.95E-16 | mdtC | 120 | B7L9U9 | transmembrane transport; xenobiotic efflux | transport | NA |
| BPAMKIAM_ | 1.631197663 | 2.49E-07 | ttgF | 120 | Q9KWV3 | efflux pump | transport | PA14_31920 |
| BPAMKIAM_ | 1.022887055 | 0.028040091 | fhuA | 120 | P06971 | ferrichrome outer membrane transporter; phage receptor; siderophore uptake | signalling | NA |
| BPAMKIAM_ | -1.064248073 | 6.82E-05 | NA | 120 | NA | NA | NA | PA14_34050 |
| BPAMKIAM_ | -1.294262111 | 1.31E-10 | NA | 120 | NA | NA | NA | PA14_34070 |
| BPAMKIAM_ | 1.13692632 | 0.003700916 | NA | 120 | NA | NA | NA | PA14_34490 |
| BPAMKIAM_ | 1.445918653 | 9.43E-08 | cmpB | 120 | Q55106 | transmembrane transport; SAPI excision | transport | PA14_34520 |
| BPAMKIAM_ | 1.137867644 | 0.00091736 | NA | 60 | NA | NA | NA | PA14_53150 |
| BPAMKIAM_ | 1.293022843 | 0.000725663 | NA | 120 | NA | NA | NA | PA14_35570 |
| BPAMKIAM_ | 1.204710599 | 3.69E-07 | mltF | 60 | NA | membrane bound murein; flagella construction | motility | PA14_36200 |
| BPAMKIAM_ | 1.707569624 | 2.52E-14 | mltF | 120 | NA | membrane bound murein; flagella construction | motility | PA14_36200 |
| BPAMKIAM_ | -1.020822807 | 9.59E-11 | NA | 120 | NA | NA | NA | PA14_36470 |
| BPAMKIAM_ | 1.1583614 | 5.18E-09 | NA | 35 | NA | NA | NA | PA14_36560 |
| BPAMKIAM_ | 1.311388046 | 0.00078505 | ligD | 120 | Q9I1X7 | NHEJ protein | nucleotide_binding | PA14_36910 |
| BPAMKIAM_ | 1.058363511 | 6.96E-08 | NA | 120 | NA | NA | NA | PA14_39090 |
| BPAMKIAM_ | 1.124419469 | 1.59E-11 | NA | 60 | NA | NA | NA | PA14_39090 |
| BPAMKIAM_ | -1.067595193 | 3.83E-09 | NA | 120 | NA | NA | NA | PA14_39270 |
| BPAMKIAM_ | 1.101983038 | 3.21E-05 | metE | 120 | P25665 | methyl group transfer | metabolism | PA14_39590 |
| BPAMKIAM_ | 1.191478537 | 0.038992177 | aroH | 120 | P80574 | aromatic amino acid biosynthesis | metabolism | NA |
| BPAMKIAM_ | -1.187850062 | 7.96E-07 | NA | 120 | NA | NA | NA | PA14_40100 |
| BPAMKIAM_ | 1.355412025 | 0.007673684 | rutR | 120 | P0ACU2 | transcriptional regulator; represses degradation of pyrimide and purine | metabolism | PA14_40380 |
| BPAMKIAM_ | 1.098442641 | 0.039598909 | rfnT | 120 | A6X7E7 | riboflavin transmembrane transport | transport | PA14_40620 |
| BPAMKIAM_ | 1.083175103 | 0.006871102 | NA | 120 | NA | NA | NA | PA14_40780 |
| BPAMKIAM_ | 1.50704939 | 0.005297368 | yfcA | 120 | P0AD30 | membrane transport | transport | PA14_40940 |
| BPAMKIAM_ | 1.156998741 | 4.86E-32 | NA | 120 | NA | NA | NA | PA14_43550 |
| BPAMKIAM_ | -1.031199915 | 1.95E-37 | ccoN | 120 | D9IA43 | cytochrome c oxidase | metabolism | NA |
| BPAMKIAM_ | -1.455329918 | 1.61E-08 | NA | 120 | NA | NA | NA | PA14_44390 |
| BPAMKIAM_ | -1.087262834 | 1.60E-22 | ccoP | 120 | D9IA45 | cytochrome c oxidase | metabolism | PA14_44360 |
| BPAMKIAM_ | 4.872301493 | 4.40E-78 | mdtJ | 120 | P69212 | spermidine export protein | transport | PA14_44520 |
| BPAMKIAM_ | 2.688084983 | 3.28E-16 | mdtJ | 60 | P69212 | spermidine export protein | transport | PA14_44520 |
| BPAMKIAM_ | 1.334359438 | 0.000134353 | mdtJ | 35 | P69212 | spermidine export protein | transport | PA14_44520 |
| BPAMKIAM_ | 4.87649411 | 3.53E-28 | mdtI | 120 | P69210 | spermidine export protein | transport | PA14_44530 |
| BPAMKIAM_ | -1.063814363 | 0.00190587 | fabR | 60 | NA | repressor of fabA and fabB | metabolism | PA14_44540 |
| BPAMKIAM_ | -1.168947815 | 1.65E-07 | fabR | 120 | NA | repressor of fabA and fabB | metabolism | PA14_44540 |
| BPAMKIAM_ | -2.129942302 | 0.000134353 | NA | 35 | Q9I3H5 | oxidoreductase | metabolism | PA14_44560 |
| BPAMKIAM_ | -1.727341696 | 0.000431222 | NA | 60 | Q9I3H5 | oxidoreductase | metabolism | PA14_44560 |
| BPAMKIAM_ | -1.342358185 | 1.06E-08 | uacT | 120 | Q46821 | uric acid transport | transport | PA14_44950 |

| | | | | | | | | |
|---|---|---|---|---|---|---|---|---|
| BPAMKIAM_ | 1.746864017 | 0.00063265 | NA | 120 | NA | NA | NA | PA14_45470 |
| BPAMKIAM_ | 1.094533451 | 6.31E-24 | ylxH | 35 | NA | flagellum site determinant | motility | PA14_45640 |
| BPAMKIAM_ | 1.59304542 | 3.26E-25 | flhF | 35 | NA | flagellar biosynthesis | motility | PA14_45660 |
| BPAMKIAM_ | 1.142924667 | 1.07E-14 | flhF | 60 | NA | flagellar biosynthesis | motility | PA14_45660 |
| BPAMKIAM_ | 1.857222847 | 2.46E-43 | flhA | 35 | NA | flagellar rod protein | motility | PA14_45680 |
| BPAMKIAM_ | 1.334324242 | 1.09E-28 | flhA | 60 | NA | flagellar rod protein | motility | PA14_45680 |
| BPAMKIAM_ | 1.086301458 | 1.23E-09 | fliN | 35 | NA | flagellar moto switch | motility | PA14_45790 |
| BPAMKIAM_ | 1.174923142 | 3.28E-34 | NA | 120 | NA | NA | NA | PA14_45830 |
| BPAMKIAM_ | 1.314504563 | 1.49E-27 | NA | 60 | NA | NA | NA | PA14_45830 |
| BPAMKIAM_ | -1.069510947 | 0.01140196 | NA | 60 | NA | NA | NA | PA14_46540 |
| BPAMKIAM_ | -1.04260299 | 6.96E-08 | NA | 120 | NA | NA | NA | PA14_46540 |
| BPAMKIAM_ | -1.033737539 | 0.039163764 | NA | 60 | NA | NA | NA | PA14_46550 |
| BPAMKIAM_ | -1.028619612 | 1.36E-07 | NA | 120 | NA | NA | NA | PA14_46550 |
| BPAMKIAM_ | -1.141910502 | 6.30E-05 | napB | 120 | NA | nitrate metabolism | metabolism | PA14_49260 |
| BPAMKIAM_ | -1.128860657 | 3.22E-16 | napC | 120 | NA | nitrate metabolism | metabolism | PA14_49270 |
| BPAMKIAM_ | 1.691820512 | 2.17E-13 | fliJ | 35 | NA | flagellar protein chemotaxis | motility | PA14_50080 |
| BPAMKIAM_ | 1.366872188 | 2.72E-07 | fliJ | 60 | NA | flagellar protein chemotaxis | motility | PA14_50080 |
| BPAMKIAM_ | 1.328154333 | 4.76E-07 | fliI | 60 | NA | flagellar protein | motility | PA14_50100 |
| BPAMKIAM_ | 1.461162189 | 2.36E-08 | fliI | 35 | NA | flagellar protein | motility | PA14_50100 |
| BPAMKIAM_ | 1.17153518 | 4.58E-06 | NA | 35 | NA | NA | NA | PA14_50110 |
| BPAMKIAM_ | 1.397326769 | 5.00E-37 | fliG | 35 | NA | flagellar motor switch | motility | PA14_50130 |
| BPAMKIAM_ | 1.690740752 | 2.13E-48 | NA | 35 | NA | NA | NA | PA14_50140 |
| BPAMKIAM_ | 1.12702555 | 1.24E-23 | NA | 60 | NA | NA | NA | PA14_50140 |
| BPAMKIAM_ | 1.072981562 | 5.02E-05 | fliE | 35 | NA | flagellar hook associated | motility | PA14_50160 |
| BPAMKIAM_ | 1.804241108 | 3.05E-24 | atoC | 60 | NA | response to spermidine motility chemotaxis and flagellum regulation | motility | PA14_50180 |
| BPAMKIAM_ | 2.182598141 | 1.93E-17 | atoC | 35 | NA | response to spermidine motility chemotaxis and flagellum regulation | motility | PA14_50180 |
| BPAMKIAM_ | 1.016185503 | 2.85E-24 | NA | 60 | NA | NA | NA | PA14_50340 |
| BPAMKIAM_ | 1.262878895 | 4.09E-27 | NA | 60 | NA | NA | NA | PA14_50360 |
| BPAMKIAM_ | 1.043370799 | 5.51E-25 | NA | 35 | NA | NA | NA | PA14_50360 |
| BPAMKIAM_ | 1.281123889 | 5.71E-11 | NA | 35 | NA | NA | NA | PA14_50380 |
| BPAMKIAM_ | 1.405569625 | 7.89E-18 | NA | 60 | NA | NA | NA | PA14_50380 |
| BPAMKIAM_ | 1.065002375 | 1.49E-10 | flgI | 120 | NA | flagellar rod protein | motility | PA14_50410 |
| BPAMKIAM_ | 1.204051232 | 5.72E-05 | flgI | 35 | NA | flagellar rod protein | motility | PA14_50410 |
| BPAMKIAM_ | 1.503268278 | 2.08E-12 | flgI | 60 | NA | flagellar rod protein | motility | PA14_50410 |
| BPAMKIAM_ | 1.676232555 | 2.08E-12 | flgH | 60 | NA | flagellar rod protein | motility | PA14_50420 |
| BPAMKIAM_ | 1.577401053 | 1.98E-10 | flgH | 35 | NA | flagellar rod protein | motility | PA14_50420 |
| BPAMKIAM_ | 1.269951549 | 6.01E-10 | flgG | 35 | NA | flagellar rod protein | motility | PA14_50430 |
| BPAMKIAM_ | 1.423850357 | 1.46E-15 | flgG | 60 | NA | flagellar rod protein | motility | PA14_50430 |
| BPAMKIAM_ | 1.498064847 | 2.80E-26 | flgF | 60 | NA | flagellar rod protein | motility | PA14_50440 |
| BPAMKIAM_ | 1.280184239 | 1.22E-21 | flgF | 120 | NA | flagellar rod protein | motility | PA14_50440 |
| BPAMKIAM_ | 1.240885682 | 2.37E-27 | flgE | 60 | NA | flagellar hook associated | motility | PA14_50450 |
| BPAMKIAM_ | 1.395180803 | 2.45E-16 | flgD | 60 | NA | flagellar rod protein | motility | PA14_50460 |
| BPAMKIAM_ | 1.031690981 | 0.002344433 | flgC | 60 | NA | flagellar rod protein | motility | PA14_50470 |
| BPAMKIAM_ | 1.353172571 | 1.21E-12 | flgB | 60 | NA | flagellar rod protein | motility | PA14_50480 |
| BPAMKIAM_ | 1.086537276 | 0.000211569 | baeR | 120 | P69229 | transcriptional regulator; responds to membane stress; links to CRISPR | transcriptional_regulat | PA14_52250 |
| BPAMKIAM_ | 1.282146537 | 0.000462698 | baeR | 60 | P69229 | transcriptional regulator; responds to membane stress; links to CRISPR | transcriptional_regulat | PA14_52250 |
| BPAMKIAM_ | -1.080980824 | 0.002940596 | NA | 120 | P32382 | NADH oxidase | metabolism | PA14_53400 |
| BPAMKIAM_ | -1.110443863 | 0.036822144 | NA | 60 | NA | NA | NA | PA14_28680 |

| | | | | | | | | |
|---|---|---|---|---|---|---|---|---|
| BPAMKIAM_ | -1.060375447 | 4.37E-06 | NA | 60 | NA | NA | NA | PA14_55750 |
| BPAMKIAM_ | -1.123868098 | 2.38E-07 | NA | 120 | NA | NA | NA | PA14_55750 |
| BPAMKIAM_ | -1.1016342 | 3.76E-05 | NA | 35 | NA | NA | NA | PA14_55750 |
| BPAMKIAM_ | -1.105292811 | 2.32E-08 | NA | 120 | NA | NA | NA | PA14_55790 |
| BPAMKIAM_ | -1.046628384 | 7.52E-12 | NA | 120 | NA | NA | NA | PA14_55940 |
| BPAMKIAM_ | -6.086510847 | 3.42E-20 | NA | 120 | NA | NA | NA | PA14_56750 |
| BPAMKIAM_ | -5.574445463 | 7.70E-19 | argO | 120 | NA | arginine export | transport | PA14_56770 |
| BPAMKIAM_ | 1.102864217 | 5.13E-05 | NA | 60 | NA | NA | NA | PA14_56830 |
| BPAMKIAM_ | 1.199956023 | 8.48E-09 | NA | 120 | NA | NA | NA | PA14_56830 |
| BPAMKIAM_ | 1.279287927 | 5.23E-05 | NA | 120 | NA | NA | NA | PA14_56840 |
| BPAMKIAM_ | 1.109353032 | 0.003669209 | NA | 60 | NA | NA | NA | PA14_56840 |
| BPAMKIAM_ | 1.148770548 | 6.76E-06 | NA | 120 | NA | NA | NA | PA14_56850 |
| BPAMKIAM_ | -1.093148534 | 0.01615761 | NA | 35 | NA | NA | NA | PA14_57640 |
| BPAMKIAM_ | -1.181562117 | 2.59E-06 | NA | 60 | NA | NA | NA | PA14_57640 |
| BPAMKIAM_ | 1.303923547 | 5.67E-31 | cysNC | 120 | NA | sulfate transport | transport | PA14_57710 |
| BPAMKIAM_ | 1.345191748 | 8.43E-08 | NA | 60 | NA | NA | NA | PA14_58560 |
| BPAMKIAM_ | 1.444799439 | 6.71E-14 | NA | 120 | NA | NA | NA | PA14_58560 |
| BPAMKIAM_ | 1.128692086 | 0.002795044 | NA | 120 | NA | NA | NA | PA14_58580 |
| BPAMKIAM_ | 1.368636291 | 0.019314339 | esiB | 120 | NA | immunoglobulin binding protein; inhibits ROS | metabolism | PA14_58600 |
| BPAMKIAM_ | -1.164357601 | 0.009899312 | NA | 60 | NA | NA | NA | PA14_59630 |
| BPAMKIAM_ | -1.177290379 | 2.97E-14 | NA | 120 | NA | NA | NA | PA14_61380 |
| BPAMKIAM_ | 1.035314352 | 0.044226138 | NA | 120 | NA | NA | NA | PA14_63880 |
| BPAMKIAM_ | -1.467974094 | 2.31E-05 | NA | 120 | NA | NA | NA | PA14_65840 |
| BPAMKIAM_ | 1.123506304 | 0.031702472 | NA | 120 | NA | NA | NA | PA14_65940 |
| BPAMKIAM_ | 2.039194822 | 9.91E-51 | ynfM | 120 | P43531 | transmembrane protein | transport | PA14_66510 |
| BPAMKIAM_ | 1.021919301 | 5.78E-07 | NA | 35 | NA | NA | NA | PA14_67440 |
| BPAMKIAM_ | 1.084988561 | 0.00137536 | NA | 120 | NA | NA | NA | PA14_68440 |
| BPAMKIAM_ | 1.000979304 | 0.001794574 | NA | 60 | NA | NA | NA | PA14_68890 |
| BPAMKIAM_ | 1.126540701 | 2.32E-19 | NA | 120 | NA | NA | NA | PA14_68900 |
| BPAMKIAM_ | 1.397794222 | 1.59E-11 | NA | 60 | NA | NA | NA | PA14_68900 |
| BPAMKIAM_ | 1.172371885 | 6.96E-07 | aspA | 60 | P0AC38 | aspartate ammonia-lyase | metabolism | PA14_71650 |
| BPAMKIAM_ | 1.454660705 | 1.98E-18 | aspA | 120 | P0AC38 | aspartate ammonia-lyase | metabolism | PA14_71650 |
| BPAMKIAM_ | -1.046449467 | 2.41E-05 | NA | 120 | NA | NA | NA | PA14_72060 |
| BPAMKIAM_ | 1.423668598 | 3.31E-06 | ivy | 35 | Q9HXB1 | inhibitor of vertebrate lysozyme | stress | PA14_72360 |
| BPAMKIAM_ | 1.413515077 | 6.27E-16 | ivy | 60 | Q9HXB1 | inhibitor of vertebrate lysozyme | stress | PA14_72360 |
| BPAMKIAM_ | 1.318105322 | 0.000360099 | NA | 35 | NA | NA | NA | PA14_72370 |
| BPAMKIAM_ | 1.551554114 | 2.62E-05 | kgtP | 120 | P0AEX3 | cation symporter; membrane transport | transport | PA14_72960 |
| BPAMKIAM_ | 1.657123321 | 5.50E-05 | kgtP | 60 | P0AEX3 | cation symporter; membrane transport | transport | PA14_72960 |
